# Active Drug-Using Women Use Female-Initiated Barrier Methods to Reduce HIV/STI Risk: Results from a Randomized Trial

**DOI:** 10.1155/2013/768258

**Published:** 2013-09-23

**Authors:** Erica Gollub, Elena Cyrus-Cameron, Kay Armstrong, Tamara Boney, Delinda Mercer, Danielle Fiore, Sumedha Chhatre

**Affiliations:** ^1^Department of Epidemiology, Robert Stempel College of Public Health and Social Work, Florida International University, 11200 SW 8th Street, Miami, FL 33199, USA; ^2^Center for Research on U.S. Latino HIV/AIDS and Drug Abuse (CRUSADA), Florida International University, Miami, FL 33199, USA; ^3^Drexel Hill, PA 19026, USA; ^4^Center for Studies on Addiction, Department of Psychiatry, University of Pennsylvania, Philadelphia, PA 19104, USA; ^5^Regional West Medical Center, Scottsbluff, NE 69361, USA; ^6^HIV/AIDS Prevention Research Division, Department of Psychiatry, University of Pennsylvania, Philadelphia, PA 19104, USA

## Abstract

*Background*. We tested an original, woman-focused intervention, based on body empowerment, and female-initiated barrier methods, including the female condom (FC) and cervical barriers. *Methods*. Eligible women were >= 18 years of age, HIV seronegative, and active drug users, reporting 30% or greater unprotected sex acts. Both controls (C) and intervention (I) participants received enhanced HIV/STI harm reduction counseling. I participants underwent 5 additional weekly group sessions. We compared change in frequency of unprotected vaginal intercourse across arms at 12 months. *Results*. Among 198 enrolled women, over 95% completed followup. Two-thirds were African-American; most of them used crack, had a primary partner, and reported sex exchange. In paired *t*-tests from baseline to followup, the frequency of unprotected vaginal sex dropped significantly for I (primary *P* < 0.00, nonprimary *P* < 0.002) and C (primary *P* < 0.008, nonprimary *P* < 0.000) arms with all partners. The difference in change across arms was of borderline significance for primary partner (*P* = 0.075); no difference was seen for nonprimary partner (*P* = 0.8). Use of male condom and FC increased with both partner types over time, but more consistently among I women. Conclusion: The “value-added” impact of the intervention was observed mainly with primary partners. Body knowledge with routine FC counseling should be incorporated into interventions for drug-using women.

## 1. Introduction

Women have among the highest rates of new HIV infections globally and the rate of new infections has not lessened in recent years [1–4]. African Americans account for 66% of new HIV infections among all US women [5]—over 5 times their proportion representation (12%) in the population. One in 30 African-American women can expect to acquire HIV infection during their lifetime [6]; the vast majority of these are through heterosexual intercourse.

Crack-using African-American women are a distinct, hard-to-reach population for whom empowerment to reduce HIV/STI risk takes on additional complexities due to the entangled dependencies of drugs, partnerships with men (who are often drug involved) for material support for women and their children, and constant threat of violent reprisal [4, 7–9]. Concurrent infection with STI is considered to be endemic among African-American, drug-using, HIV+ women [10].

Women's biological inequality in vulnerability to sexually transmitted infection (STI) can be addressed with greater availability and use of male and female condoms (FC). In addition to challenges in the diffusion of available protection technologies, however, gender inequity in sexual relationships, limited bargaining power of women complicated by threats of violence, and insufficient resource commitment to health issues among minorities and the poor all contribute to women's continuing entrenchment of HIV/AIDS in the United States.

The FC is the only female-initiated protection method that protects against HIV/STI and unwanted pregnancy at a level equivalent to a male condom (MC; [11]). Full integration of the FC has lagged far behind in HIV prevention efforts [12–14]. For example, “best evidence” interventions for US populations at high risk [15] infrequently (4 of 14 studies) included information and counseling on the FC, although most studies were targeted to heterosexual risk groups. Formal CDC documents on voluntary counseling and testing (VCT) do not specifically refer to FC as integral to the counseling approach. Adoption of the FC into routine counseling has been inconsistent and lacking energy.

Effective interventions targeted to drug-using women to reduce sexual risk are few in number and, as pointed out by Wechsberg et al. [16], have rarely involved follow-up intervals of longer than 6 months. Studies with short-term followup evaluating effective intervention approaches have included two woman-focused interventions: the Co-Op model [17] as well as the NIDA standard model tested by Sterk and colleagues [18], as against two enhanced models.

 The Womens' Co-Op Intervention is a brief 4-session intervention with mixed individual and group sessions, based on empowerment and feminist theory, encouraging women to understand their HIV risks in the context of substance abuse and effects on personal power and vulnerability to victimization [17]. Numerous adaptations have been conducted since the first trial of the intervention, which resulted in its recognition as a best evidence intervention [15], based on results from 6-month followup. Long-term followup (4 years) of 60% of the original study population, however, found evidence of differential attrition favoring the retention of the high-risk participants and lack of long-term sustained salutary effects observed at 6 months [16].

Sterk and colleagues [18] followed 333 mainly crack-using women who were randomly assigned to three different conditions. With 96% retention at 6 months, positive change was demonstrated in drug use behavior and sexual risk behavior, as well as drug-related sexual risk behavior such as sex trade. However, no differences among the NIDA standard and woman-focused interventions were found. The enhanced interventions were based on five behavioral theories with a gender- and culture-specific focus. All conditions involved access to prevention methods and included practice of negotiation and protection techniques and technologies.

The behavioral intervention tested here (*“BestBET”*) integrated elements from three existing theories—the Theory of Gender and Power [19], Community Empowerment Theory [20], and Risk Reduction/Harm Reduction Theory—as well as an original theory of “body empowerment.” The latter draws heavily from feminist health principles espoused widely in the 1970s in such works as *Our Bodies, Ourselves* [21]. The BestBET intervention was initially developed as a multiple session, clinic-based intervention among HIV+ women [22], and has evolved through a series of studies on diverse populations of high-risk women [23–25]. Increased body knowledge appeared to facilitate use of women's barrier methods in these studies because risk behavior declined.

We believed that active crack/polydrug users would attend a relatively long, multisession intervention with women-focused content despite the inherent physical and mental challenges involved. We posited that the woman-specific intervention themes would have cross-cutting relevance and produce positive behavioral change among this population at very high sexual risk for HIV/STI. 

In this paper, we describe a randomized controlled trial of an intervention (“BestBET”) from a sample of 198 drug-using women with high-risk sexual behavior who were followed for 12 months.

## 2. Methods

### 2.1. Intervention

The feminist health model as applied to HIV underscores the need for holistic education about reproductive organs and genitals, rather than a narrow focus on HIV. Our intervention included session content on normal female anatomy, the menstrual cycle, pregnancy, menopause, STI signs and symptoms, female cancers, benefits of screening (mammography and pap smear), and common surgeries. The body empowerment approach posits that increased knowledge, sense of ownership, and pride in the body (“body knowledge”) are an independent pathway to self-esteem and to self-efficacy for protection behavior—use of female-initiated barriers, MC negotiation with partners, and refusal of unsafe sex. Thus, we introduced to intervention women the entire range of female methods thought possibly to reduce risk of STI/HIV—female condom, diaphragm, cervical cap, and spermicides (the study was initiated prior to the findings concerning vaginal irritation with nonoxynol-9 [26]; we counseled women to abandon use of spermicides for disease protection following publication of the findings)—arranged “hierarchically” according to what was known about protection level [23] in a harm reduction approach to increasing protection behavior (i.e., “something is better than nothing”). We also focused on the need for female solidarity and mutual support. These four pillars—body knowledge, access to female initiated methods, emphasis on harm reduction, and a setting promoting female solidarity—provided the intervention core components. We integrated content on female and male barriers in each intervention session, focusing on practical applications of the FC with diverse partners, including paid sexual transactions (e.g., “cheeking” or oral application of MC without partner initiation) concomitant with drug use (e.g., inserting an FC before getting high), and situations of potentially violent partners (inserting cervical barrier with spermicide for discreet risk reduction). Women tried the female barriers at home and shared their experiences at the following group session, where troubleshooting for problems encountered in the initial adoption period was facilitated by a trained counselor.

The impact of basic body education coupled with access to a full range of female barrier options on decreasing sexual risk behavior has not, to our knowledge, been evaluated in active substance users. For this trial, we enhanced certain elements and adapted the study to target out-of-treatment drug users. We added a session dedicated to intimate partner violence (for paying, nonprimary, and primary partners) including a module on basic self-defense techniques and exit strategies and use of protection methods in the context of forced sex. The intervention was held at a university-operated, community storefront site with a long history of provision of HIV counseling and testing in a poor, inner city neighborhood. The intervention was culturally specific and woman focused. 

The elements of the intervention were (1) small groups with interactive counseling; (2) trained and certified near-peer community counselors with a history of substance abuse and in recovery for at least 2 years; (3) role play and rehearsal, (4) multimedia educational approaches, such as videos, audio tapes, brochures, posters, and anatomical models; (5) active referrals to local service organizations; (6) access to HIV testing; and (7) training of CBO staff for community capacity building. Audio tapes of diverse risk vignettes based on interviews with the target population and accompanying illustrated handouts were prepared by an outside nonprofit health organization. We collaborated with a nearby branch of a national family planning organization to facilitate diaphragm and cervical cap fittings and offered patient advocate support to accompany study participants to clinic appointments.

### 2.2. Eligibility and Recruitment

To be eligible for the BestBET study, we required women to (1) be 18 years of age or older, (2) be HIV seronegative, (3) report 30% or more unprotected vaginal or anal sex acts over the preceding three months (averaged over all partners), (4) report not currently being in drug user treatment other than with Methadone, and (5) report that heroin or cocaine were either injected, snorted, or smoked at least 12 times during the same three-month interval. HIV serostatus was determined using ELISA with confirmatory Western Blot. Women were excluded for psychiatric problems or if they had been in drug treatment for more than 6 months. Because changing drug use behavior was not an explicit study objective, we did not exclude women from attending sessions if they were “high” except if such use disrupted group dynamics.

Eligible women were recruited in Philadelphia between November 2001 and August 2003, with the use of a mobile outreach van staffed with trained interviewers and harm reduction counselors. The van was parked in designated, high-risk areas known for crack-selling and smoking activity. Interested women gave the first written consent to be administered a 20-minute, confidential prescreening assessment interview that included demographic and behavioral risk items as well as to provide locator information. The second screening assessment was conducted at a community storefront site that served as an information and referral center for drug users. Potential participants completed a second written informed consent procedure for the full study. Study compensation for each visit was $25. Recruitment and study methods have, in part, been presented in a prior publication [27].

This study was approved by and conducted in compliance with the Institutional Review Board of the University of Pennsylvania. All enrolled women provided written informed consent.

### 2.3. Data Collection and Retention Efforts

Participants completed a baseline risk assessment instrument delivered via audio, computer-assisted self-interview (a-CASI) targeting sexual and drug user behavior over prior 6 months. Partner types were divided into primary and nonprimary. Additional baseline data were collected in a face-to-face interview including the following: demographic and reproductive health history, history of STI, health care insurance, and types and location of health care services used in past 6 months. A knowledge quiz on the reproductive system and disease prevention methods was also administered [28].

Following baseline data collection, all women received enhanced HIV and STI harm-reduction counseling that exceeds standard guidelines for HIV voluntary counseling and testing (VCT); both the MC and FC were taught. Participants then underwent testing for HIV/STI. At the posttest session, we randomized HIV-negative women to intervention or control group. We counseled women with STI on treatment locations and required these women to bring written confirmation of treatment for formal enrollment. We provided HIV-positive women with referrals for further counseling and care but they were excluded from this study.

Women randomized to the intervention arm were invited back for five, 3-hour, weekly group sessions within 2 months of enrollment and were contacted for 6- and 12-month reunion sessions at community sites. Control participants received limited case management and the offer of free MCs and FCs, but no other proactive study intervention. At 6- and 12-month following enrollment, all assessments were repeated at the community sites or other locations when necessary.

Masters' level interviewers were trained and certified in VCT and in standardized interviewing techniques. Participant contact information was updated at least quarterly. Intensive retention efforts (phone, mailed correspondence, and in person) were used to follow participants considering their mobility, including community outreach, visits to known crack houses and other hang-outs, and hospital and prison outreach. Retention challenges were discussed weekly in study meetings. Using multiple methods and extensive efforts to locate hard-to-reach participants, the retention rate was 95% at 12 months.

### 2.4. Statistical Analysis

To test for differences between the control and intervention groups at baseline, chi-square for dichotomous variables and independent sample *t*-test for continuous variables were conducted for demographic variables and background characteristics. Univariate analyses including frequencies and percentages were completed to describe the total sample and its subsets at baseline and 12-month follow-up. 

Our main outcome variable, frequency of unprotected vaginal intercourse (monthly), is the focus of this paper. Data for this analysis were derived from a series of questions delivered via a-CASI, asking first, the total frequency of acts during the interval (vaginal, anal, and oral) followed by the frequency of acts protected by MC or FC, diaphragm, cervical cap, or spermicide (these latter three related only to vaginal sex). The remainder was defined as unprotected vaginal sex acts. Protected and unprotected vaginal sexual acts were calculated at baseline and at 12-month followup for two types of partners: primary partners and HIV-negative nonprimary partners. The number of HIV-positive nonprimary partners was prohibitively small to include for analysis. A primary (male) partner was described as “someone you have lived with or have seen a lot, and to whom you have felt a special emotional commitment.” nonprimary partners were defined as “other male partner(s) (not your primary or main partner).” If a woman answered that she did not have vaginal sex in the prior interval (this was only possible at followup) or did not have that type of partner, she was considered as having zero frequency of unprotected acts. For this study we used an intent-to-treat analysis with the inclusion of women who may not have been sexually active during followup and/or may not have had a primary or nonprimary sexual partner.

Pairwise analysis was performed on the outcome variable separately for primary and nonprimary partners from baseline to followup, for each intervention arm, which included generating a paired sampled *t*-test (*P* < 0.05 was considered significant). For comparison of intervention versus control arms, first a “change” variable was calculated in the mean frequency of unprotected acts between baseline and followup. The change was then compared across arms. Either parametric (Student's *t*-test) or nonparametric tests (Mann-Whitney *U* test) were used to assess statistical significance, depending on the normality of distribution. All analyses were conducted using SPSS version 19.0 (SPSS, Inc., Chicago, Illinois).

## 3. Results

### 3.1. Sample Demographics and Risk Behavior

**Figure 1:**
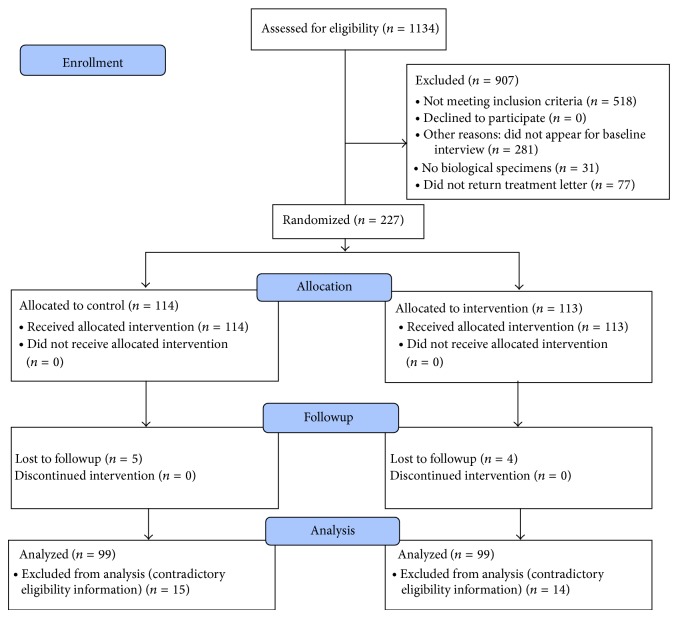
CONSORT 2010 flow diagram.

A total of 1134 women were prescreened at the van. Of the 616 eligible women, 304 (49%) successfully completed the baseline interviews and specimen collection and were invited to participate in the BestBET study. Among eligible women completing baseline interviews (*n* = 304), six percent were HIV positive, and overall STI prevalence was 49% [25]. Of these women, 227 (75%) returned for enrollment procedures and were randomized (113 as intervention subjects and 114 as controls). Twenty-nine women were later excluded based on contradictory information related to eligibility criteria (see Figure 1). A slightly greater percentage of eligible women who completed baseline interviews, as compared to the final study group, reported drug injection (49% versus 42%) and somewhat fewer had health insurance (65% versus 70%). A smaller percentage reported a primary partner (78% versus 83%). Other demographic and behavioral differences were less significant or nonexistent. 

The majority of the study participants were African-American (see Table 1). Their mean age was just under 40 years. A majority of participants were unemployed with nearly two-thirds reporting use of food stamps. Approximately half had a high school diploma or equivalent educational certificate. Most women had a history of drug treatment, with crack/rock cocaine and marijuana as the drugs of choice. Over 40% of women in both arms reported ever injecting drugs, mainly heroin. Most participants also had a history of sex exchange. A substantial number reported recent jail or shelter stay.

 Most participants (C 79.8%, I 85.9%) had a primary male sex partner, and more than half (C 56.6%, I 60.6%) had both a recent nonprimary male sex partner(s) and a primary partner. Women with primary partners reported substantial levels of recent (past 6 months) intimate partner violence: 21% had partners who had made threats on their life, 34% had been forced to have vaginal or anal sex without a condom, 25% had been punched or hit with something that hurt, and 11% had consulted medical care because of a fight (data not shown). No significant differences across arms were found.

### 3.2. Paired Analyses: Baseline to Followup, Control, and Intervention Arms

#### 3.2.1. Unprotected Vaginal Sex Acts

At baseline, the overall mean frequency of unprotected vaginal sex acts (all enrolled women) with primary partners in the past 6 months was 34.3 (median = 12), and with nonprimary, HIV-negative partners was 22.7 acts (median 0). At 12-month followup, the overall mean frequency of unprotected vaginal sex acts (all enrolled women) with primary partners was 15. 6 (median 0); with nonprimary HIV-negative partners the follow-up mean frequency was 2.9 acts (median 0). A total of 189 women completed assessments at 12-month followup (retention rate: 95.4%). 

#### 3.2.2. Primary Partner

For the paired baseline-to-followup comparisons, among *controls*, for primary partner, baseline frequency was 32.7 acts; at 12 months this was reduced to 19.3 acts (paired *t*-test, *P* = 0.008; see Table 2). Among *intervention* women, mean of unprotected vaginal acts at baseline for primary partner was 35.9 and at follow-up was 12.3 (paired *t*-test, *P* = 0.000).

#### 3.2.3. Nonprimary Partners

For *control* women with nonprimary HIV partners, mean baseline frequency of unprotected vaginal acts was 21.6 acts; at 12-month followup frequency was 2.5 (paired *t*-test, *P* = 0.000). Among *intervention* women, frequency of unprotected vaginal acts with non- primary HIV-partners at baseline was 23.8, and at 12-month followup was 3.5 (paired *t*-test, *P* = 0.002).

A statistical test of the change over time (i.e., reduction in unprotected acts), per partner type, compared across arms, indicated borderline significance for primary partners (*P* = 0.075), favoring the intervention arm. For nonprimary partners, no statistical difference could be detected between the change observed across the two arms from baseline to followup (*P* = 0.80).

### 3.3. Use of MC and FC

Proportions of women reporting MC use were greater in both arms in cross-sectional analyses comparing baseline to followup. MC use with primary partners was lower than with nonprimary partners across both arms, at both baseline and followup (Table 3). Intervention women reported MC use with primary partners more frequently at followup but the difference across arms was not statistically significant for either primary or nonprimary partners. MC use with nonprimary partner was reported by two-thirds (I) and three-quarters (C) of women; the higher rates in I women at follow-up were achieved despite lower usage at baseline with nonprimary partners (I versus C). Use of the FC was also substantially higher at followup than at baseline in both arms. The lowest proportions of women reporting FC use at followup were C women with nonprimary partners (7%). Use of spermicide and cervical barriers was also greater at followup compared with baseline for all women except C women with their nonprimary partners. Very few protected acts involved spermicide use alone and only 1 woman reported use of a cervical barrier unaccompanied by either MC or FC use. Thus, followup method use as compared with baseline method use appeared to increase the least when considering the subgroup “control women-nonprimary partners.” For I women, by contrast, there were consistent and substantial differences when comparing baseline to followup, with both partner types and across all methods.

## 4. Discussion 

The sample of active, drug-using women in this study demonstrated large changes in risk behavior over 12-month follow-up. Frequency of unprotected sex acts dropped significantly for both study arms, across both partner types. The difference in frequency of unprotected sex for both study arms was modified by partner type. For primary partners, that difference was substantial, although not entirely reaching statistical significance. Statistical significance would likely have been realized with greater numbers due to the large variability in the outcome measure. For nonprimary partners, there was no observed difference across the study arms; women in both arms reported substantial increases in protection. Our findings agree with those of others indicating that use of MC is less frequent with a primary partner than with nonprimary partners [29]; nevertheless, the results with primary partner for intervention women are encouraging as these partners are often considered to pose a greater risk of STI/HIV transmission to drug-using women [8]. Although both types of counseling reduced unprotected acts, the “value-added” impact of the group-based intervention appeared to reside mainly with primary partners.

In our study, FC use also increased in both arms, with greater and more uniform increases among I women. In particular, at followup, fewer C women used FC with nonprimary partners, with proportions that were significantly different from I women. We have previously shown that introduction to the FC and other female protection methods promoted MC negotiation and possibly a greater rejection of unprotected sex [23]. These findings taken together suggest that our group-based intervention treatment was superior in encouraging behavior change. Prior studies among drug-using women have often suffered from short follow-up periods [16]. Our 95% retention rate at 12 months allowed increased confidence in our findings. 

Study participants who used FC found the method sufficiently practical to use with primary and nonprimary partners. There has been a tendency to discount female barrier methods as impractical and unacceptable though no wide-scale evidence supports this contention and indeed there is evidence to the contrary [13, 14]. Women have potentially more control over FC (versus MC) use but its impact will only be realized with strong promotional programs and quality counseling including outreach activities to men wherever possible [30] and easy access to continuing supplies, to ensure adoption and maintenance of the behavior [12]. The FC should be a regular component of VCT, formalized into easily accessible counseling guidelines and promoted widely. A separate analysis of knowledge changes with this intervention [28] suggests that a key component of FC adoption is empowerment of women through better knowledge of their bodies. Such education should be incorporated routinely into future interventions and programs for drug-using women—including, importantly, woman-focused drug treatment programs, still few in number and often lacking a reproductive health approach [8].

Our body empowerment approach and counselor training supplied women with key elements to boost the use of female-initiated methods, including cervical barriers and devices that are important and relevant to drug-using women [31] but poorly promoted and underresearched as a dual protection method [32].

### 4.1. Limitations

This study had several limitations. Study participants were selected on the basis of specific criteria drawn up to concentrate a high level of drug-related and sexual risk. They agreed and were expected to attend five, relatively long, intervention sessions and return for follow-up assessments. Other drug-using populations with a lower risk profile or less availability for study procedures might have demonstrated different results. All self-reported behavior is subject to social desirability bias. Nevertheless, we used a-CASI [33], an established technique to increase the validity of reporting of sensitive behavior in numerous populations. Additionally, there was high variability in frequency of reported unprotected acts at both time points. These considerations tend to mitigate concerns about reporting bias, though there is no way of eliminating this possibility. Comparisons of FC and MC use proportions were cross-sectional due to high levels of changes in partner and sexual activity. The last data were collected in 2004, potentially raising concerns about relevance. Drug-using women continue to be at extremely high risk for HIV/STI, with large demonstrated racial disparities, despite successful inroads made for other at-risk populations in the interim, such as non-injection-using men who have sex with men [34]; additional approaches are still urgently needed [35]. Finally, we did not employ an “attention control” arm, as we designed two alternative counseling approaches that could be used in the field; thus changes over time cannot be attributed to the intervention procedures with complete confidence and may be due in part to the additional time duration spent with counselors.

Of concern, we may well have underestimated the impact of our intervention as compared to MC counseling only, which is practiced widely. We did not find it ethical to withhold FC from control participants. Also, our analysis was based on an intention-to-treat approach; women assigned to the intervention arm did not necessarily complete all five sessions. The sample size may have been too small to detect some changes that were statistically significant—for example, the differences within main partner across intervention and control arms. Finally, contamination of the message across study arms was certainly possible, given the relatively small geographic recruitment area and overlapping social networks; this too would have acted to minimize observed differences across the study arms.

## 5. Conclusion

The HIV primary prevention landscape is changing, with oral preexposure prophylaxis (PrEP) as well as postexposure prophylaxis (PEP) now documented to be valuable tools in the prevention mix and potentially applicable to drug-using populations [36]. The potential for reducing sexual risk with vaginal PrEP for women is still unclear, with conflicting and recently disappointing results regarding the efficacy of vaginal tenofovir [37, 38]. A daily, voluntary application of an antiviral vaginal compound may suffer from poor adherence; long-term intact options may be considerably more effective. As additional topical microbicidal compounds complete testing, including microbicidally impregnated vaginal devices [39], the counseling on the use of such methods, especially among women at high risk such as IDU and NIDU women, will become more routine. Our results suggest that active drug-using women at high risk of sexually transmitted HIV/STI will partake of such counseling and capitalize on expanded access to these technologies.

## Figures and Tables

**Table 1:** Baseline characteristics by randomization group∗.

	Control (*n* = 99)	Intervention (*n* = 99)
Demographic		
Age	39.6 ± 7.9	39.5 ± 8.7
Race		
White	29.0	26.0
African American	63.0	68.0
Latino	10.0	5.0
Other	10.0	5.0
SES/health care		
GED or HS diploma	51.0	52.5
Earns less than $1000 per month	77.6	82.8
Unemployed	92.8	93.9
Receives welfare	52.4	51.4
Receives food stamps	65.6	63.6
Shelter stay—past 6 months	13.2	9.2
Has regular medical provider	78.8	72.4
Has some type of health insurance	73.5	65.7
Has Medicaid	50.0	44.2
Reproductive health history/services		
Primary sexual partner only—past 6 months	79.8	85.9
Both primary and nonprimary partners—past 6 months	56.6	60.6
Ever pregnant	96.8	91.2
Mean number of live births	3.25 ± 2.2	3.0 ± 2.3
Pap smear—past 6 months	33.3	39.4
HIV test—past 6 months	32.2	42.4
HIV/STI risk indicators		
Past 6 months:		
Daily alcohol use	27.3	22.2
Daily marijuana use	12.1	8.1
Daily cocaine use	9.1	6.1
Daily crack use	56.6	46.5
Ever injected drugs	42.4	42.4
Ever exchanged sex for money	80.8	79.8
Ever exchanged sex for drugs	66.7	69.7

^*^Data: % except where mean ± SD.

**Table 2:** Paired comparisons, mean frequency of unprotected vaginal sex, baseline to followup, by partner type (*N* = 189; 94 controls, 95 intervention)∗.

Partner type	Baseline mean	12-month follow-up mean	*P* value; change∗∗ over time	*P*-value; change∗∗ across arms
Primary partner	34.3	15.6	0.000	
Control	32.7	19.3	0.008	0.075
Intervention	35.9	12.3	0.000	
Non-primary, HIV-negative partner	22.7	2.9	0.000	
Control	21.6	2.5	0.000	0.80
Intervention	23.8	3.5	0.002	

^*^Absence of acts or no partner equals zero unprotected acts (see Methods).

^**^Change defined as follow-up frequency − baseline frequency.

**Table 3:** Reported use of protection methods (% of women users) in the prior six months, at baseline and followup, by partner and study arm (cross-sectional analysis).

Primary partner	Baseline			12-month followup		
	Control (*n* = 74) *n* (%)	Intervention (*n* = 80) *n* (%)	*P *	Control (*n* = 42) *n* (%)	Intervention (*n* = 47) *n* (%)	*P *
Male condom	14 (19)	14 (18)	0.82	14 (33)	20 (42)	0.20
Female condom	3 (4)	5 (6)	0.72	12 (29)	9 (19)	0.24
Spermicide	4 (5)	6 (8)	0.72	6 (14)	10 (21)	0.42
Diaphragm/cervical cap	0	3 (4)	0.25	7	1 (2)	0.34

HIV-negative, non-primary partner	Control (*n* = 64) *n* (%)	Intervention (*n* = 55) *n* (%)		Control (*n* = 28) *n* (%)	Intervention (*n* = 24) *n* (%)	

Male condom	60 (94)	38 (69)	0.02	21 (75)	16 (67)	0.51
Female condom	5 (8)	4 (7)	0.74	2(7)	6 (24)	0.13
Spermicide	6 (9)	7 (13)	0.85	1 (4)	5 (20)	0.08
Diaphragm/cervical cap	0	2 (4)	0.47	0 (0)	1 (4)	0.46
